# Supplementary material for: Unc13A and Unc13B contribute to the decoding of distinct sensory information in Drosophila
Source: Nat Commun. 2021 Mar 26;12:1932. doi: 10.1038/s41467-021-22180-6 (PMC7997984; doi:10.1038/s41467-021-22180-6)
Supplement: Supplementary file 1 — Supplementary Information [file 41467_2021_22180_MOESM1_ESM.pdf]

# Unc13A and Unc13B contribute to the decoding of distinct sensory information in *Drosophila*

Atefeh Pooryasin, Marta Maglione, Marco Schubert, Tanja Matkovic-Rachid, Sayed-mohammad Hasheminasab, Ulrike Pech, André Fiala, Thorsten Mielke, Stephan J. Sigrist

## Supplementary Information

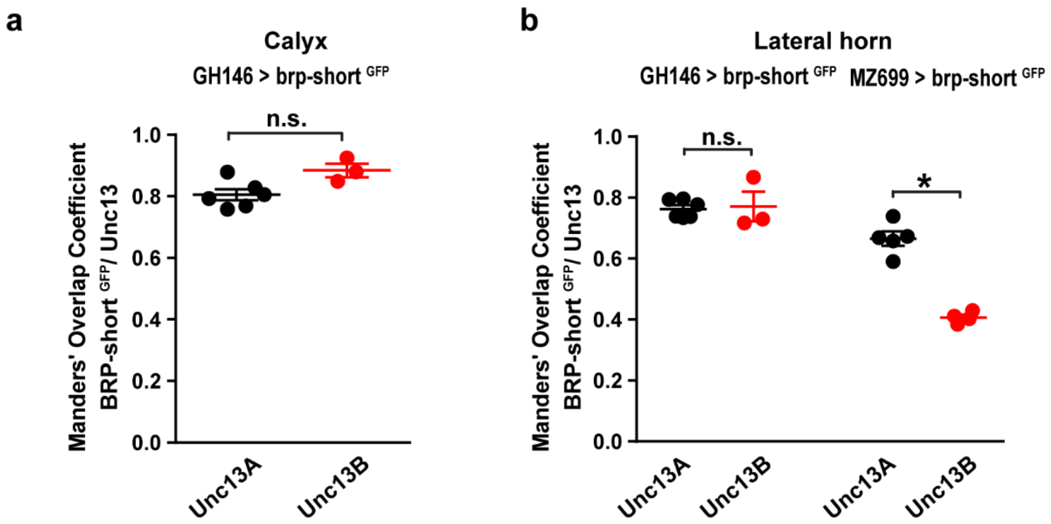


**Supplementary Figure 1.** Unc13A and Un13B localization relative to BRP-short$^{GFP}$ at active zones of ePNs and iPNs pre-synapses in MB calyx and lateral horn. **a** Mander's overlap coefficient for BRP-short$^{GFP}$ with Unc13A (black) or Unc13B (red) in the MB calyx in flies expressing UAS: brp-short$^{GFP}$ in ePNs (n=3-6). **b** Mander's overlap coefficient for BRP-short$^{GFP}$ with Unc13A (black) or Unc13B (red) in the lateral horn region in flies expressing UAS: brp-short$^{GFP}$ under control of GH146-Gal4 (ePNs, n=3-6) or Mz699-Gal4 (iPNs, n=4-5). Values represent mean ± SEM. All p-values were calculated via two-tailed Mann-Whitney U test. n.s., not significant ($p>0.05$), *$p\leq0.05$. Source data including the exact sample sizes and the p values are provided as a Source Data file.

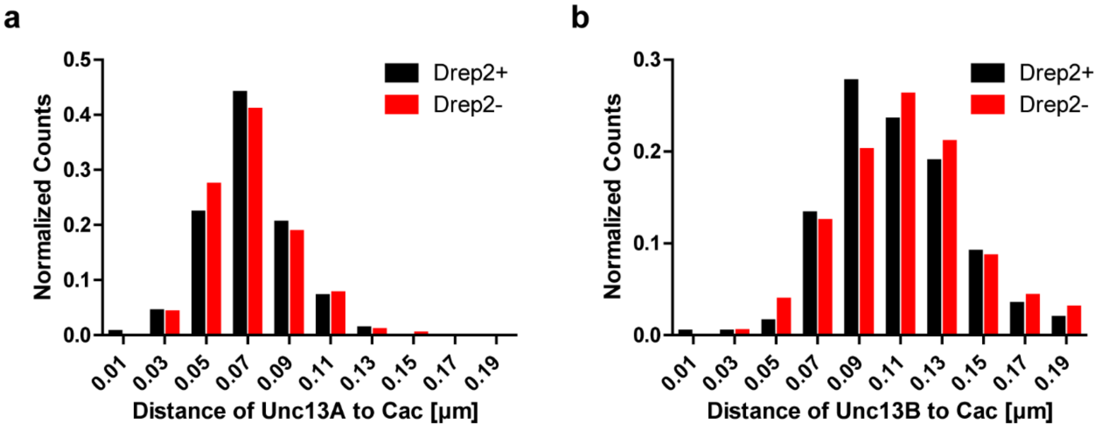


**Supplementary Figure 2**. Time gated STED analysis of Unc13A and B nano-distribution at LH synapses**. a** Frequency histogram for all Unc13A-sfCac distances found for Drep2-positive (n=323 synapses, 7 brains) and Drep2-negative synapses (n=315 synapses, 7 brains; p>0.999, two samples Kolmogorov-Smirnov test), in the LH. Counts were normalized to obtain a probability density function with an integral equal 1. **b** Frequency histogram for all Unc13B-sfCac distances found for Drep2-positive (n=264 synapses, 7 brains) and Drep2-negative synapses (n=233 synapses, 7 brains; p=0.9883, two samples Kolmogorov-Smirnov test), in the LH. Counts were normalized to obtain a probability density function with an integral equal 1. Source data are provided as a Source Data file.

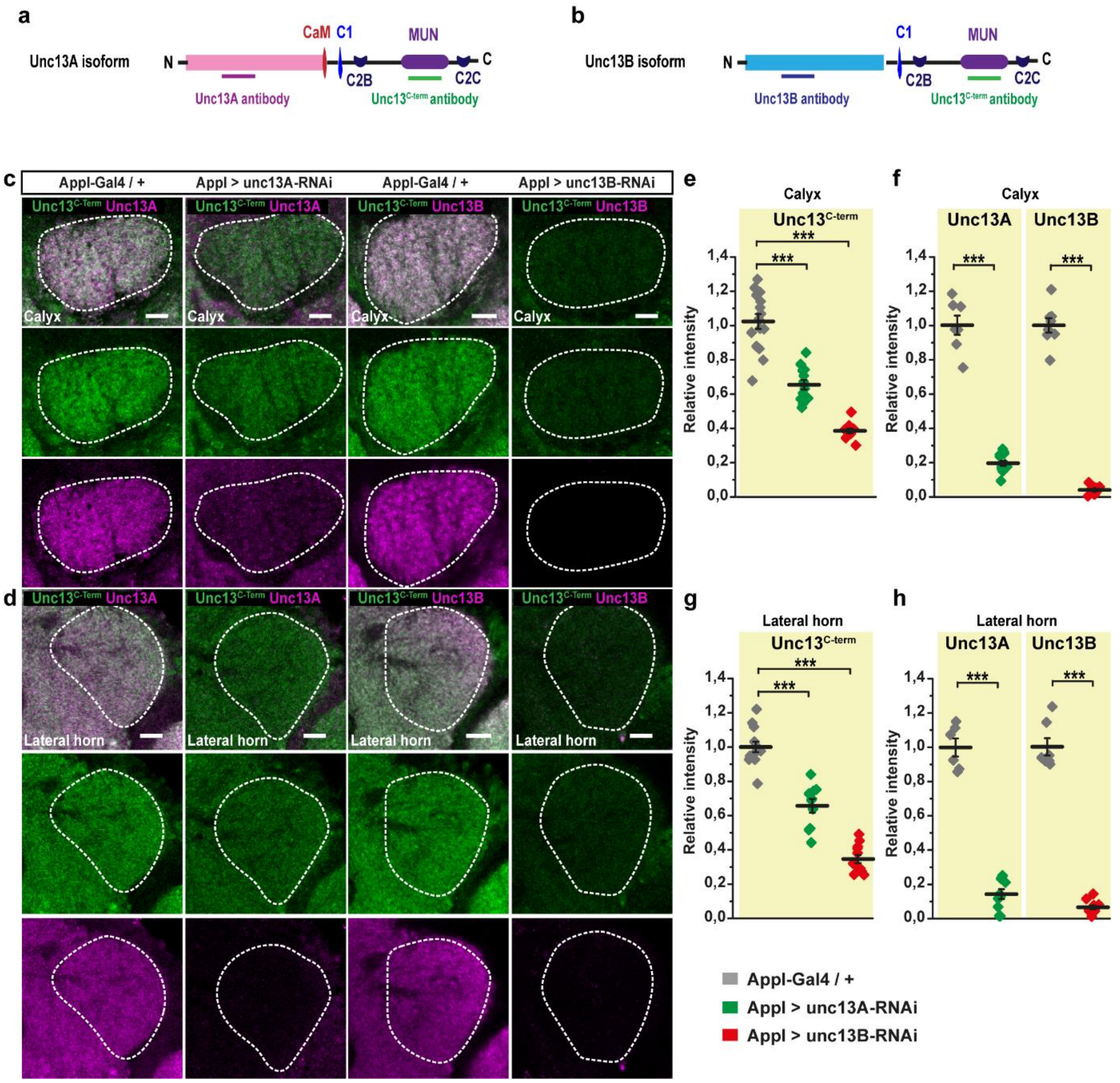


**Supplementary Figure 3.** Relative levels of Unc13A versus Unc13B in calyx and lateral horn. **a, b** Schematics of Unc13A **(a)** and Unc13B **(b)** constructs indicating CaM, calmodulin; C1, C2B, C2C, and the MUN domain. The Unc13$^{C\text{-term}}$ antibody and isoform specific Unc13A and Unc13B antibody epitopes are indicated. **c, d** Confocal images from the calyx **(c)** and lateral horn **(d)** regions in animals expressing UAS: unc13A-RNAi under control of Appl-Gal4, compared to heterozygous driver line, Appl, immunostained against Unc13$^{C\text{-term}}$ (green) and Unc13A (magenta) or in animals expressing UAS: unc13B-RNAi under control of Appl-Gal4, compared to heterozygous driver line, Appl, immunostained against Unc13$^{C\text{-term}}$ (green) and Unc13B (magenta). Scale bars: 10 µm. **e, g** Expression level of Unc13$^{C\text{-term}}$ protein in calyx **(e)** and lateral horn **(g)** regions in animals expressing UAS: unc13A-RNAi or UAS: unc13B-RNAi under control of Appl-Gal4, compared to heterozygous driver line, Appl. The remaining

Unc13$^{C\text{-term}}$ signal in *unc13A* knockdown animals which represents the Unc13B level in calyx (0.63.± 0.02) and in lateral horn (0.65 ± 0.03) were measured. The remaining Unc13$^{C\text{-term}}$ signal in *unc13B* knockdown animals which represents Unc13A level in calyx (0.36 ± 0.03) and in lateral horn (0.34 ± 0.03) were measured (n = 10-15). P-values were calculated via one-way ANOVA with the Bonferroni multiple comparison *post hoc* test (***p≤0.001). **f, h** Expression level of Unc13A or Unc13B protein in calyx **(f)** and lateral horn **(h)** regions in animals expressing UAS: unc13A-RNAi or UAS: unc13B-RNAi under control of Appl-Gal4, compared to heterozygous driver line, Appl. The values show a significant reduction of Unc13A in *unc13A* knockdown group (green) compared to the control group (gray plot). The level of Unc13B significantly reduced in *unc13B* knockdown group (red plot) compared to the control group (gray plot) (n= 6-13). P-values were calculated via two-sample t-test (***p≤0.001). Values represent mean ± SEM. (***p≤0.001). Source data including the exact sample sizes and the p values are provided as a Source Data file.

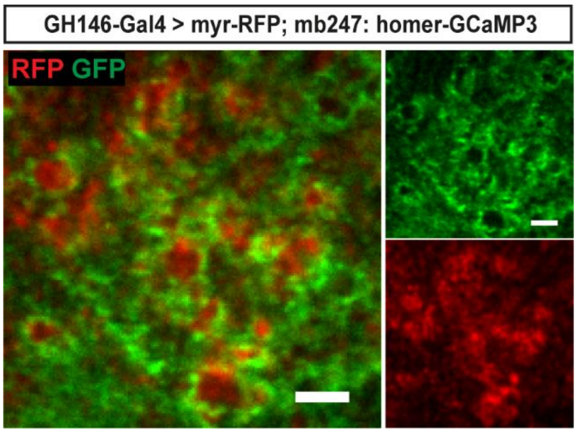


**Supplementary Figure 4.** Expression pattern of homer-GCaMP3 in the MB Calyx. Visualizing microglomerular structures in the calyx region of animals expressing homer-GCaMP3 sensor in MB postsynaptic terminals under control of mb247 promoter (mb247: homer-GCaMP3) and myr-RFP in ePNs under the control of GH146-Gal4. Green: anti-GFP immunostaining against homer-GCaMP3; Red: anti-RFP immunostaining in presynaptic terminals of ePNs (n=3, the experiment was performed once). Scale bars: 2 μm.

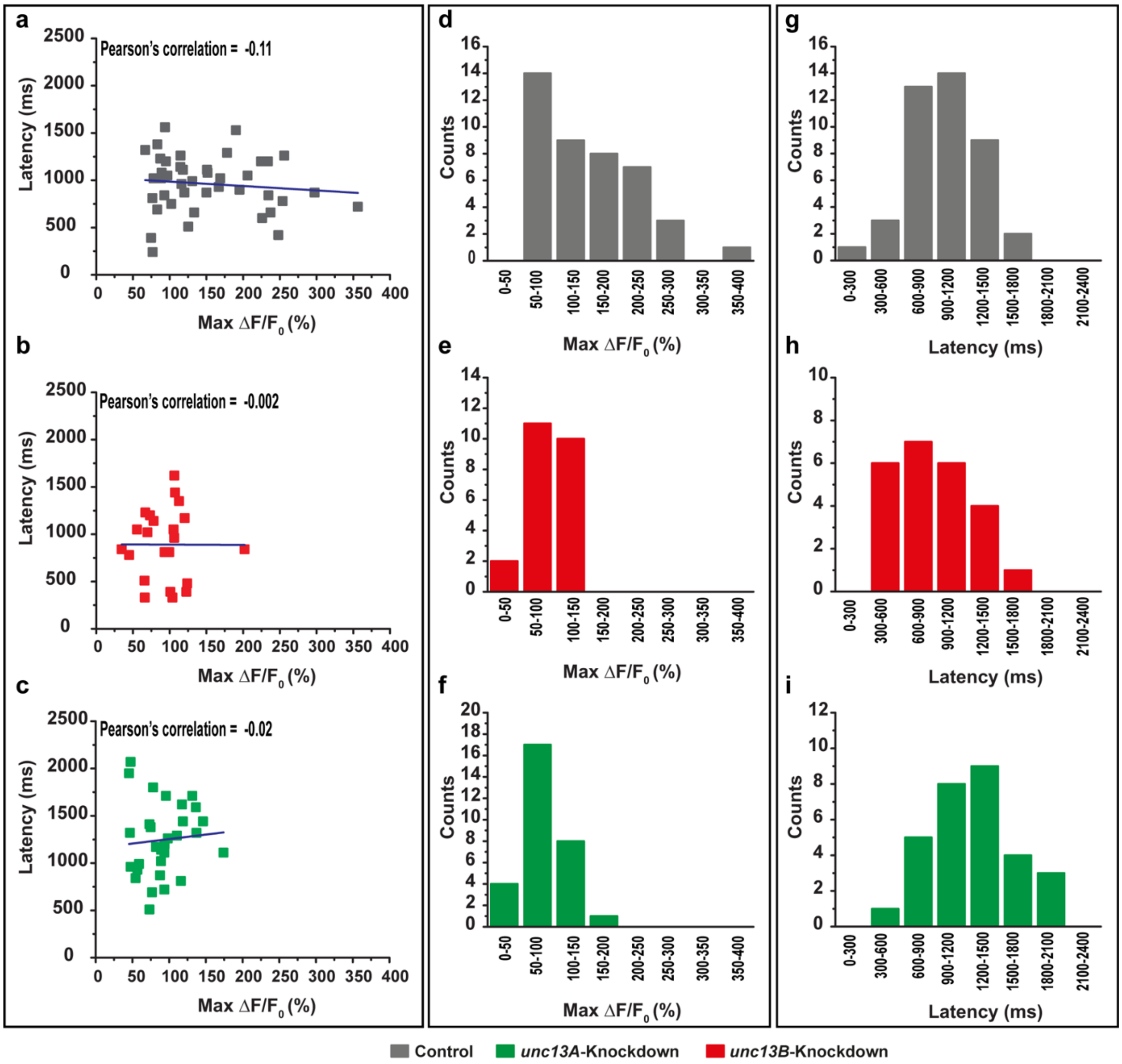


**Supplementary Figure 5.** Release kinetics (latencies) and $Ca^{2+}$ signal amplitudes across different odor responsive microglomeruli. **a-c** The maximum amplitude ($\Delta F/F_0$) plotted against time to peak (latency) for each responsive microglomerulus for control (*GH146-Gal4 / +; mb247: homer-GCaMP3 /* +), *unc13B*-knockdown (*GH146* > *unc13B-RNAi; mb247: homer-GCaMP*3) and *unc13A*-knockdown (*GH146* > *unc13A-RNAi; mb247: homer-GCaMP3)* groups. The Pearson's correlation scores show no correlation between amplitude and the latency in any group. **d-i** Frequency histogram for maximum amplitude ($\Delta F/F_0$) **(d-f)** and time to peak (latency) **(g-i)** for control (*GH146-Gal4 / +; mb247: homer-GCaMP3 /* +), *unc13B*-knockdown (*GH146* > *unc13B-RNAi; mb247: homer-GCaMP*3) and *unc13A*-knockdown (*GH146* > *unc13A-RNAi; mb247: homer-GCaMP3)* groups across different odor responsive microglomeruli. Odor stimulation: 4-methylcyclohexanol. n=24-42 microglomeruli, 8-12 animals. Experiment was performed four times. Source data including the exact sample sizes are provided as a Source Data file.

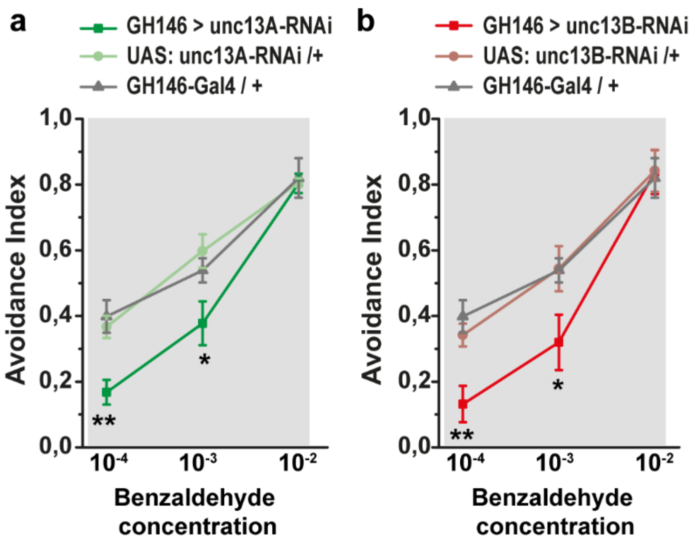


**Supplementary Figure 6.** Olfactory behavior toward different concentrations of aversive odor after down regulation of *unc13A* or *unc13B* in ePNs Knockdown of *unc13A* **(a)** or *unc13B* **(b)** under control of GH146-Gal4 reduces the performance index of animals toward aversive odor benzaldehyde in low concentrations (diluted $10^{-4}$ and $10^{-3}$ in paraffin oil, n=9-12). No significant change was observed for high concentration of benzaldehyde (diluted $10^{-2}$ in paraffin oil, n=4-6). All p-values were calculated via one-way ANOVA with the Bonferroni multiple comparison *post hoc* test (*p≤0.05, **p≤0.01). Graphs indicate mean ± SEM. Source data including the exact sample sizes and the p values are provided as a Source Data file

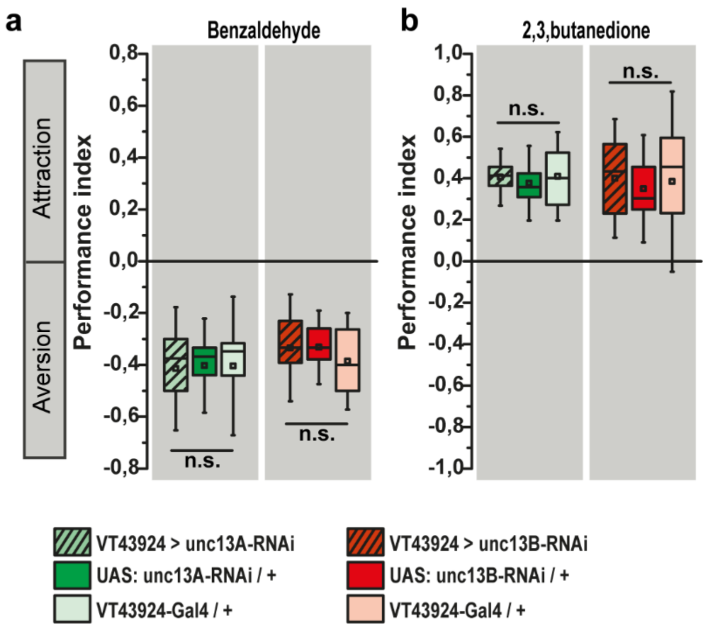


**Supplementary Figure 7.** Role of APL neurons in olfactory behavior. **a, b**. *unc13A* knockdown or *unc13B* knockdown in APL neurons using VT43924-Gal4 driver line has no significant effect in performance index of animals toward aversive (benzaldehyde n=13-22) and appetitive (2, 3-butanedione, n=14-22) odors**.** P-values were calculated via one-way ANOVA with the Bonferroni multiple comparison *post hoc* test, n.s., not significant (p>0.05). Box plots indicate means (black squares), medians (middle lines), 25$^{th}$ and 75$^{th}$ percentile ranges (boxes), and SD ranges (whiskers). Source data including the exact sample sizes and the p values are provided as a Source Data file.

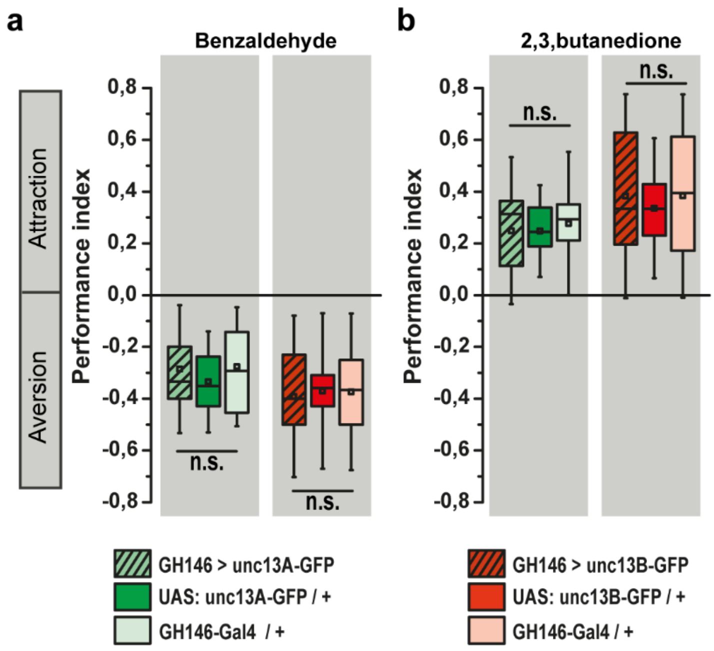


**Supplementary Figure 8.** Overexpression of the unc13A or unc13B in ePNs. **a, b**. *unc13A-GFP* or *unc13B-GFP* overexpression in ePNs neurons using GH146-Gal4 driver line has no significant effect in performance index of animals toward aversive (benzaldehyde n=14-17) and appetitive (2, 3-butanedione, n=16-19) odors. P-values were calculated via one-way ANOVA with the Bonferroni multiple comparison *post hoc* test, n.s., not significant (p>0.05). Box plots indicate means (black squares), medians (middle lines), 25$^{th}$ and 75$^{th}$ percentile ranges (boxes), and SD ranges (whiskers). Source data including the exact sample sizes and the p values are provided as a Source Data file.

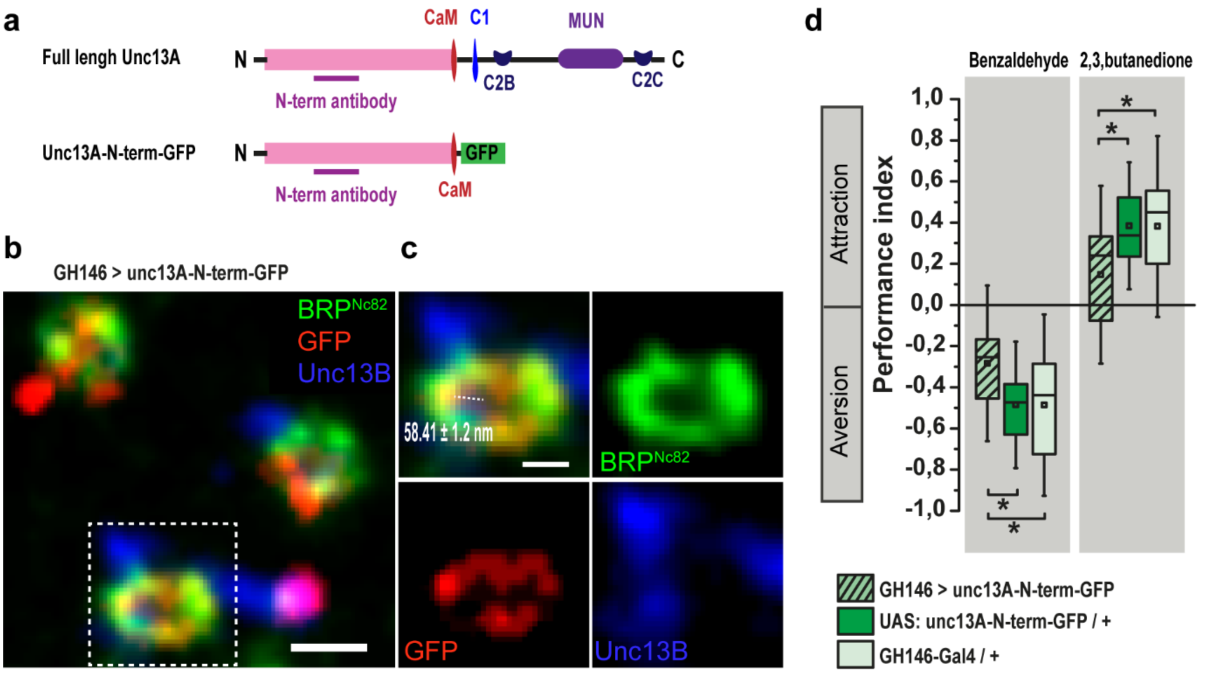


**Supplementary Figure 9.** Disruption of Unc13A dependent release by overexpressing *unc13A*-N-terminal fragment in ePNs. **a** Schematics of full length Unc13A (upper) and *unc13A*-N-term-GFP (lower) constructs indicating CaM, calmodulin; C1, C2B, C2C, and the MUN domain and the GFP tag. **b, c** Triple channel time gated STED (gSTED) images of GFP distribution (red) relative to BRP$^{NC82}$ (green) and Unc13B (blue) in calyx synapses in animals expressing UAS: *unc13A*-N-term-GFP under control of GH146-Gal4.Insets **(c)** show a planar BRP ring. GFP was found to be at 58.4 ± 1.2 nm (Mean ± SEM) to the center of the BRP ring (n=4 brains, 244 synapses, experiment was performed once). Scale bars: **b** 200 nm, **c**. 100 nm White square indicates the magnified region. **d** Overexpression of *unc13A*-N-terminal fragment in ePNs under control of GH146-Gal4 leads to a significant reduction in performance index in response to both appetitive (2, 3-butanedione, n=20-22) and aversive (benzaldehyde, n=21-28) odors. All p-values were calculated via one-way ANOVA with the Bonferroni multiple comparison *post hoc* test (*p≤0.05). Box plots indicate means (black squares), medians (middle lines), 25$^{th}$ and 75$^{th}$ percentile ranges (boxes), and SD ranges (whiskers). Source data including the exact sample sizes and the p values are provided as a Source Data file.

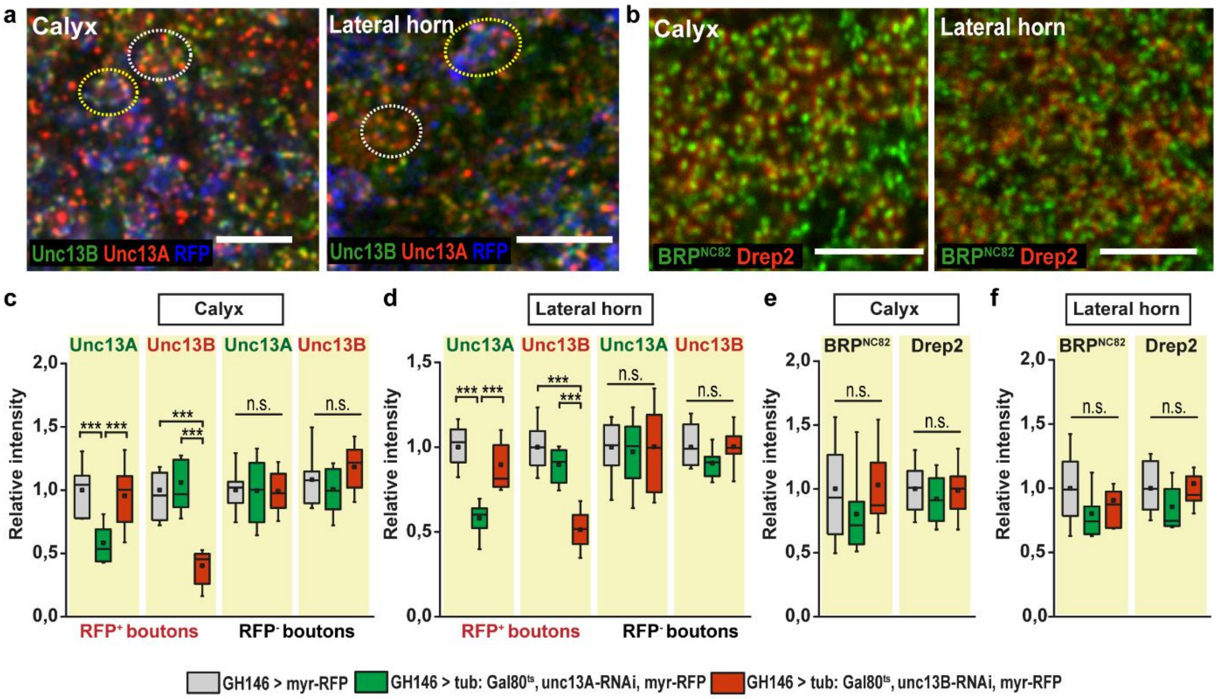


**Supplementary Figure 10.** Restriction of the *unc13A* or *unc13B* knockdown to adult stages using a tub: Gal80$^{ts}$ approach **a** Confocal images from the calyx and the LH of animal expressing myr-RFP in ePNs under the control of GH146-Gal4 (GH146 > myr-RFP) immunostained against Unc13A (red), Unc13B (green) and RFP (blue). The yellow and white circles indicate the RFP$^{+}$ and RFP$^{-}$ boutons respectively. Scale bars: 5µm. **b** Confocal images from the calyx and the LH represent anti-BRP (green) and anti-Drep2 (red) immunostaining. Scale bars: 5 µm. **c** Expression level of Unc13A and Unc13B proteins in the RFP$^{+}$ and RFP$^{-}$ calycal boutons. The box plot shows a significant reduction of Unc13A in animals expressing unc13A-RNAi, myr-RFP (green plot) compared to the control group (gray plot) while no change was observed for Unc13B level. The level of Unc13B significantly reduced in animals expressing unc13B-RNAi, myr-RFP (red plot) compared to the control group (gray plot) while no change was observed for Unc13A level. In RFP$^{-}$ calycal boutons, the level of Unc13A and Unc13B remains unchanged for all groups. **d** Expression level of Unc13A and Unc13B proteins in the RFP$^{+}$ and RFP$^{-}$ boutons of the LH. The box plot shows a significant reduction of Unc13A in animals expressing unc13A-RNAi, myr-RFP (green plot) compared to the control group (gray plot) while no change was observed for Unc13B levels. The levels of Unc13B significantly decreased in animals expressing unc13B-RNAi, myr-RFP (red plot) compared to the control group (gray plot) while no change was observed for Unc13A levels. In RFP$^{-}$ boutons of the LH, the levels of Unc13A and Unc13B remains unchanged for all groups. **e, f** Quantification of BRP$^{NC82}$ and Drep2 levels in the calyx (**e**) and the LH (**f**) boutons reveals no change

among different groups. Box plots indicate means (black squares), medians (middle lines), 25th and 75th percentile ranges (boxes), and 10–90% ranges (whiskers). n=12-15. All p-values were calculated via one-way ANOVA with the Bonferroni multiple comparison *post hoc* test (n.s., not significant ($p>0.05$), *$p\leq0.05$, **$p\leq0.01$, ***$p\leq0.001$. Source data including the exact sample sizes and the p values are provided as a Source Data file.

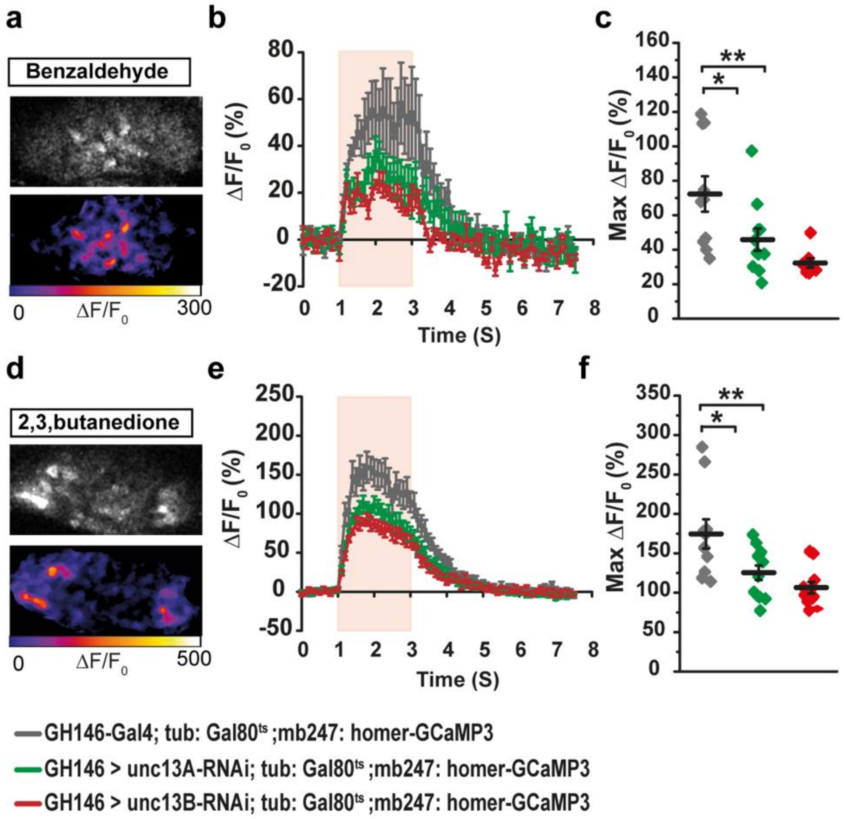


**Supplementary Figure 11.** Physiological deficit after *unc13A* or *unc13B* knockdown using the tub: Gal80$^{ts}$ approach. **a, d** Representative false color-coded two-photon images of odor-induced fluorescence changes of GCaMP3 in MB calyx (mb247: homer-GCaMP3) at the dendritic terminals of Kenyon cells. For odor stimulation, benzaldehyde **(a)** and 2, 3-butanedione **(d)** were used. **b, e** Odor-induced fluorescence change ($\Delta F/F_0$) of homer-GCaMP3 in animals expressing UAS: unc13A-RNAi (green trace) or UAS: unc13B-RNAi (red trace) in ePNs under control of GH146-Gal4 in adult stage using tub:Gal80$^{ts}$ was compared to the control group (*GH146-Gal4/ tub: Gal80$^{ts}$; mb247: homer-Gcamp3/+,* gray trace). **c, f** Maximum fluorescence changes of GCaMP3 upon odor stimulation, benzaldehyde **(c)** and 2, 3-butanedione **(f)** in animals with *unc13A* (green) or *unc13B* (red) knockdown compared to their genetic control (gray). For odor stimulation Benzaldehyde (n=8-12) and 2, 3-butanedione (n=10-12) were used. All p-values were calculated via one-way ANOVA with the Bonferroni multiple comparison *post hoc* test (*$p \leq 0.05$, **$p \leq 0.01$). All traces represent mean ± SEM of $\Delta F/F_0$% values. Error bars indicate SEM. Source data including the exact sample sizes and the p values are provided as a Source Data file.

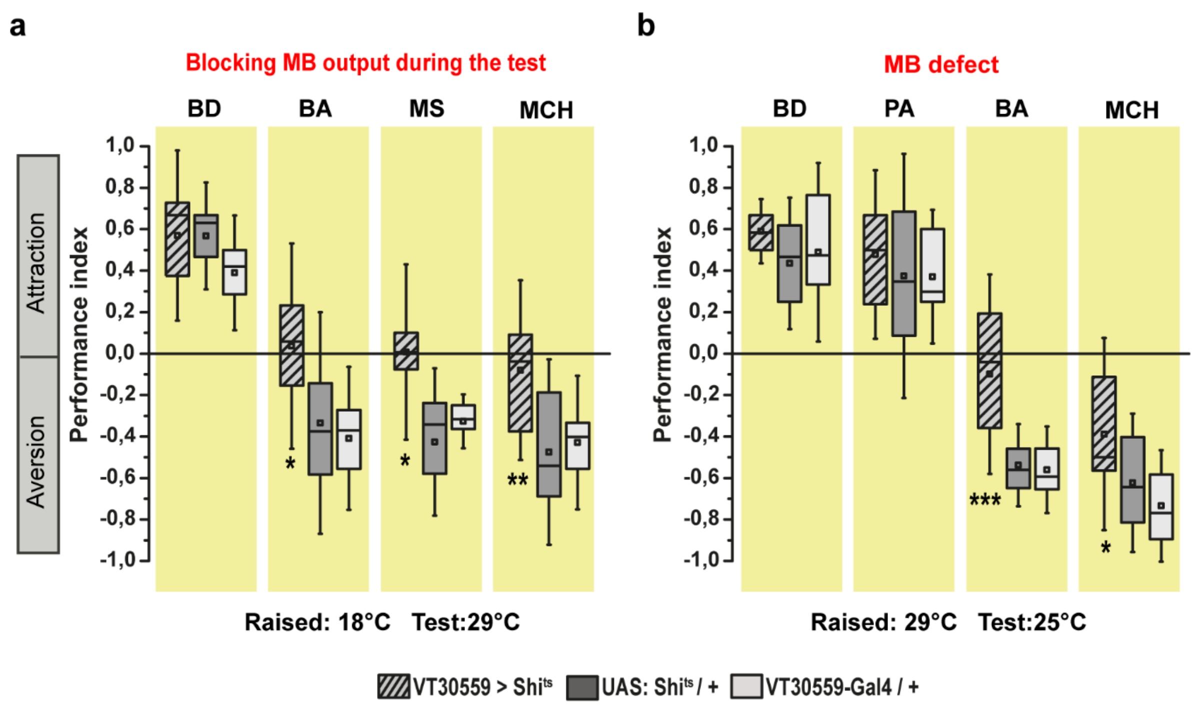


**Supplementary Figure 12.** Mushroom bodies role in olfactory coding. **a** The output of MB neurons was blocked by expressing Shi$^{ts}$ under control of VT30559-Gal4 driver line. Animals were kept at 18°C and tested at 29°C. Blocking the MB neurons at the time of test leads to a significant reduction in performance index of animals toward aversive odors (BA, n=9-12; MS, n=6 and MCH, n=8-14). No significant change was observed in VT30559> Shi$^{ts}$ animals toward appetitive odor (BD, n=13-17). **b** Chronic blocking of the output of MB neurons by expressing Shi$^{ts}$ under control of VT30559-Gal4 driver line and raising the animals at 29°C. The VT30559> Shi$^{ts}$ animals with MB defect shows a significant reduction in performance index toward aversive odors (BA, n=12 and MCH, n=15-16) and no change toward appetitive odors (BD, n=13-17 and PA, n=10-13). BA: benzaldehyde, MS: methyl salicylate, MCH: 4-methylcyclohexanol, BD: 2, 3-butanedione, PA: propionic acid. All p-values were calculated via one-way ANOVA with the Bonferroni multiple comparison *post hoc* test (**p≤0.01, ***p≤0.001). Box plots indicate means (black squares), medians (middle lines), 25$^{th}$ and 75$^{th}$ percentile ranges (boxes), and SD ranges (whiskers). Source data including the exact sample sizes and the p values are provided as a Source Data file.

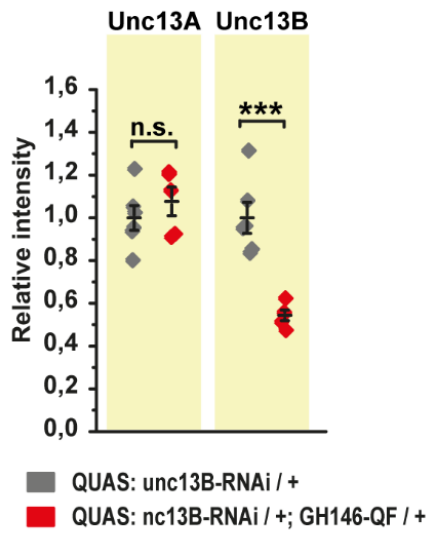


**Supplementary Figure 13.** Efficient Knockdown of *unc13B* in ePNs using QUAS/QF system. Expression level of Unc13A and Unc13B proteins in calycal boutons in animals expressing QUAS: unc13B-RNAi under control of GH146-QF, compared to the control group. The values show a significant reduction of Unc13B in *unc13B*-knockdown group (red plot) compared to the control group (gray plot) while no change was observed for Unc13A level. P-values were calculated via two-sample t-test. (n.s., not significant (p>0.05), ***p≤0.001, n= 5-6). Values represent mean ± SEM. Source data including the exact sample sizes and the p values are provided as a Source Data file.

| **Primer name** | **Primer sequence** |
|---|---|
| **UAS- gypsy F1** | CGACAAGCCGAATTGATCCACTAGAAGGCCTAATTCGGTACACTAGT |
| **UAS- gypsy R1** | CACGTGACTCGAGAAAGAGAGAGAGAAAGCTGGTACTACTAGTGTTGTTG |
| **UAS- gypsy F1** | ACACTAGTAGTACCAGCTTTCTCTCTCTCTTTCTCGAGTCACGTG |
| **UAS- gypsy R2** | AGCGTAGCGGATCCGTAAGCTTCGGCTATCGAGTCACTGAGTCCCAACGTGAAAGG |

**Supplementary Table 1**. Sequences of primers used in this study.
