# Supplementary material for: Unc13A and Unc13B contribute to the decoding of distinct sensory information in Drosophila
Source: Nat Commun. 2021 Mar 26;12:1932. doi: 10.1038/s41467-021-22180-6 (PMC7997984; doi:10.1038/s41467-021-22180-6)
Supplement: Supplementary file 3 — Reporting Summary [file 41467_2021_22180_MOESM3_ESM.pdf]

nature research

Corresponding author(s): Stephan J. Sigrist

Last updated by author(s): Feb 24, 2021

Reporting Summary

Nature Research wishes to improve the reproducibility of the work that we publish. This form provides structure for consistency and transparency in reporting. For further information on Nature Research policies, see Authors & Referees and the Editorial Policy Checklist .

Statistics

For all statistical analyses, confirm that the following items are present in the figure legend, table legend, main text, or Methods section.

n/a	Confirmed	
☐	☒	The exact sample size (n) for each experimental group/condition, given as a discrete number and unit of measurement
☐	☒	A statement on whether measurements were taken from distinct samples or whether the same sample was measured repeatedly
☐	☒	The statistical test(s) used AND whether they are one- or two-sided<br>*Only common tests should be described solely by name; describe more complex techniques in the Methods section.*
☒	☐	A description of all covariates tested
☐	☒	A description of any assumptions or corrections, such as tests of normality and adjustment for multiple comparisons
☐	☒	A full description of the statistical parameters including central tendency (e.g. means) or other basic estimates (e.g. regression coefficient) AND variation (e.g. standard deviation) or associated estimates of uncertainty (e.g. confidence intervals)
☐	☒	For null hypothesis testing, the test statistic (e.g. F, t, r) with confidence intervals, effect sizes, degrees of freedom and P value noted<br>*Give P values as exact values whenever suitable.*
☒	☐	For Bayesian analysis, information on the choice of priors and Markov chain Monte Carlo settings
☒	☐	For hierarchical and complex designs, identification of the appropriate level for tests and full reporting of outcomes
☒	☐	Estimates of effect sizes (e.g. Cohen's d, Pearson's r), indicating how they were calculated

Our web collection on statistics for biologists contains articles on many of the points above.

Software and code

Policy information about availability of computer code

Data collection	Leica,LAS X software, Leica Microsystems (LAS X 3.5.2.18963)<br>Zeiss, ZEN (Black 2.3)<br>Abberior Instruments Expert line, Imspector software (16.1.6477, Germany)
Data analysis	Fiji software (1.52P) for adjusting brightness, merging of emission channels and calculating average and maximal intensity projections across the z-axis,Line profile measurements of distances between spots. Matlab (R2016a) for correcting the potential slight movements in x-y direction for live imaging experiment. GraphPad Prism(version 7.03).

For manuscripts utilizing custom algorithms or software that are central to the research but not yet described in published literature, software must be made available to editors/reviewers. We strongly encourage code deposition in a community repository (e.g. GitHub). See the Nature Research guidelines for submitting code & software for further information.

Data

Policy information about availability of data

All manuscripts must include a data availability statement. This statement should provide the following information, where applicable:
- Accession codes, unique identifiers, or web links for publicly available datasets
- A list of figures that have associated raw data
- A description of any restrictions on data availability

All data supporting the findings of this study are provided within the paper and its supplementary information. All additional information and Drosophila lines generated for this study will be made available upon reasonable request to the corresponding author. Source data are provided with this paper.

Field-specific reporting

Please select the one below that is the best fit for your research. If you are not sure, read the appropriate sections before making your selection.

☒ Life sciences ☐ Behavioural & social sciences ☐ Ecological, evolutionary & environmental sciences

For a reference copy of the document with all sections, see nature.com/documents/nr-reporting-summary-flat.pdf

Life sciences study design

All studies must disclose on these points even when the disclosure is negative.

Sample size	Sample sizes where chosen in accordance with previous studies in the field and our observations in previous reports.<br>Gupta, V. K. et al. Spermidine Suppresses Age-Associated Memory Impairment by Preventing Adverse Increase of Presynaptic Active Zone Size and Release. PLoS biology 14, e1002563, doi:10.1371/journal.pbio.1002563 (2016).<br>Hussain, A. et al. Inhibition of oxidative stress in cholinergic projection neurons fully rescues aging-associated olfactory circuit degeneration in Drosophila. eLife 7, doi:10.7554/eLife.32018 (2018).
Data exclusions	For live calcium imaging, only samples were considered that were not moving in Z direction during imaging.
Replication	Behavioral experiments, live imaging and immunohistochemistry experiments were performed independently multiple times (overs several days, 3-4 times) under the same conditions.All attempts at replicationwere successful.
Randomization	No randomization was used and samples were determined by their genotype. Experiments performed in alternating manner between genotypes and similar number of groups for each genotype was measured in each experimental day.
Blinding	No blinding was used for performing experiments. The analysis criteria were chosen before conducting the experiment and followed strictly to minimize the bias. For behaviour experiments, all groups were prepared and treated in the same way (temperature, food, age) and strictly analyzed in an unbiased manner. For immunostainings, all genotypes were prepped in one session, stained with the same antibody solution, scanned in one session (genotypes in an alternating manner) and strictly analyzed in an unbiased manner. Live-imaging experiments were performed in an alternating fashion in regards to the genotype, aiming to gather equal data from each group in any particular day and were also strictly analyzed in an unbiased manner.

Reporting for specific materials, systems and methods

We require information from authors about some types of materials, experimental systems and methods used in many studies. Here, indicate whether each material, system or method listed is relevant to your study. If you are not sure if a list item applies to your research, read the appropriate section before selecting a response.

Materials & experimental systems

n/a	Involved in the study	
☐	☒	Antibodies
☒	☐	Eukaryotic cell lines
☒	☐	Palaeontology
☐	☒	Animals and other organisms
☒	☐	Human research participants
☒	☐	Clinical data

Methods

n/a	Involved in the study	
☒	☐	ChIP-seq
☒	☐	Flow cytometry
☒	☐	MRI-based neuroimaging

Antibodies

Antibodies used	Guinea-pig anti-Unc13A (Bohme et al., 2016). Guinea-pig anti-Unc13B (Bohme et al., 2016). Rabbit anti-Unc13B (Bohme et al., 2016). Rat anti-RFP (Chromotek, Cat# 5F8). Chicken anti-GFP (Abcam # ab13970). Mouse anti-BRP NC82 (DSHB, Cat# nc82). Rabbit anti-Drep2C-Term (Andlauer et al., 2016). rabbit anti-Unc13-C-Term (Reddy-Alla et al., 2017). Alexa Fluor 488-coupled goat anti-chicken (Invitrogen A11039). Cy3-coupled goat anti-guinea pig (Abcam, Cat# ab102370). Alexa Fluor 633-coupled goat anti-rabbit (Invitrogen, Cat# A21071). Alexa Fluor 647-coupled goat anti-guinea pig (Invitrogen, Cat# A21450). Cy3-coupled goat anti-rat (Invitrogen, Cat# A10522). Alexa Fluor 488-coupled goat anti-guinea pig (Invitrogen, Cat# A11073). Alexa Fluor 488-coupled goat anti-mouse (Invitrogen, Cat# A11029).Alexa Fluor594-coupled goat anti-guinea pig (Invitrogen, Cat# 11076). STARRED-coupled goat anti-chicken (Abberior, Cat# STRED-1005-500UG). ATTO490 LS-coupled goat anti-rabbit (Hypermol, Cat# 2309). Alexa Fluor 488-coupled goat anti-rabbit (Invitrogen, Cat# A11034). STARRED-coupled goat anti-mouse (Abberior, Cat# STRED-1001-500UG)
Validation	Primary antibodies:<br>1. chicken anti-GFP (Abcam, ab13970)-https://www.abcam.com/gfp-antibody-ab13970.html-<br>Specificity: Our GFP antibody does cross-react with the many fluorescent proteins that are derived from the jellyfish Aequorea

victoria. These are all proteins that differ from the original GFP by just a few point mutations (EGFP, YFP, mVenus, CFP, BFP etc.).
Tested applications: Suitable for: WB, ICC/IF
Yu X et al. Dorsal root ganglion macrophages contribute to both the initiation and persistence of neuropathic pain. Nat Commun 11:264 (2020).
2. Mouse anti-BRP NC82 (DSHB, Cat# nc82)-https://dshb.biology.uiowa.edu/nc82
Antigen Species: Drosophila, Positive Tested Species Reactivity: Drosophila, Mosquito. Depositors Notes: This antibody specifically labels presynaptic active zones. Recommended Applications: Immunofluorescence, Immunohistochemistry, Western Blot
White-Grindley, E. et al. Contribution of Orb2A stability in regulated amyloid-like oligomerization of Drosophila Orb2. PLoS biology 12, e1001786, doi:10.1371/journal.pbio.1001786 (2014).
3. Rat anti-RFP (Chromotek, Cat# 5F8)- https://www.chromotek.com/products/detail/product-detail/rfp-antibody-5f8/
Description: Rat monoclonal [5F8] to Red Fluorescent Proteins (RFP). Specificity: Tested on dsRed, mRFP, mCherry , mPlum, mRFPruby, mScarlet, tdTomato. For more details, please see our Fluorescent protein specificity table. Applications: Immunofluorescence: 1:1,000, ELISA: 1:1,000.

4. Guinea-pig anti-Unc13A . Guinea-pig anti-Unc13B. Rabbit anti-Unc13B :
Bohme, M. A. et al. Active zone scaffolds differentially accumulate Unc13 isoforms to tune Ca(2+) channel-vesicle coupling. Nature neuroscience 19, 1311-1320, doi:10.1038/nn.4364 (2016).

5. Rabbit anti-Unc13-C-Term:
Reddy-Alla, S. et al. Stable Positioning of Unc13 Restricts Synaptic Vesicle Fusion to Defined Release Sites to Promote Synchronous Neurotransmission. Neuron 95, 1350-1364 e1312, doi:10.1016/j.neuron.2017.08.016 (2017).

6. Rabbit anti-Drep2C-Term:
Andlauer, T. F. et al. Drep-2 is a novel synaptic protein important for learning and memory. eLife 3, doi:10.7554/eLife.03895 (2014).

Animals and other organisms

Policy information about studies involving animals; ARRIVE guidelines recommended for reporting animal research

Laboratory animals

The following fly strains were used for the different experiments:
VT43924-Gal4
Appl-Gal4
UAS: unc13A-C-term
GH146-Gal4
Mz699-Gal4
UAS:unc13A-RNAi
UAS:unc13B-RNAi
sfCacGFP
UAS:brp-shortGFP
tub:Gal80ts (BDSC #7019)
UAS: myr-RFP (BDSC #7118)
GH146-QF, QUAS:mcd8-GFP (BDSC #30038)
UAS:unc13A-NtermGFP
VT30559-Gal4 (VDRC # v206077)
20xUAS:Shi (ts)

For immunostaing and live imaging experiments female flies (5-6 day old) were used. For behavior experimnets both female and male flies (5-6 day old) were used. For Tub:Gal80(ts) experiments, the flies were 10-11 day old at the time of the experiment.

Wild animals

The study did not involve wild animals

Field-collected samples

The study did not involve collection of samples from the field

Ethics oversight

No ethical guidance or approval was obtained due to no ethical restrictions working with Drosophila melanogaster

Note that full information on the approval of the study protocol must also be provided in the manuscript.
